# Supplementary material for: COVID-19 outcomes in patients taking cardioprotective medications
Source: PLoS One. 2022 Oct 10;17(10):e0275787. doi: 10.1371/journal.pone.0275787 (PMC9550077; doi:10.1371/journal.pone.0275787)
Supplement: Attachment — Submitted filename: CVmedsCOVIDoutcomes-PLOSOne-ResponseToReviewers02-v01.docx [file pone.0275787.s002.docx]

Alexander Turchin, MD, MS

Division of Endocrinology

Brigham and Women’s Hospital

221 Longwood Avenue

Boston, MA 02115

617-732-5661

[aturchin@bwh.harvard.edu](mailto:aturchin@bwh.harvard.edu)

Manuscript # 20-1201-Orig

Masaki Mogi

Academic Editor

PLOS ONE

September 13, 2022

Dear Dr. Mogi,

We would like to thank the Editors and Reviewers for their thoughtful comments detailed in your letter from September 13, 2022. We have incorporated these suggestions in the manuscript. Below we will detail, item by item, our responses to the Editors/Reviewers’ recommendations.

REVIEWER # 2

*The authors partially answered the questions put forward by this reviewer. However, the suggestion on the use of DNA/RNA aptamers remains. The authors should better mention and discuss perspective of aptamers directed toward ACE2 (Pharmacol Res. 2022 Jan;175:105982.) and those directed toward the coronavirus spike protein (Proc Natl Acad Sci U S A. 2021 Dec 14;118(50):e2112942118.).*

RESPONSE

We have added a discussion of DNA / RNA aptamers being developed for treatment of COVID in the manuscript (in the Discussion section) as advised by the Reviewer (including the references recommended by the Reviewer).

Thank you for your comprehensive feedback, and we appreciate the opportunity to revise our manuscript. Please do not hesitate to contact us should you have any further questions.

Yours sincerely,

Alexander Turchin, MD, MS
